# The development of using function word “ *zài* ” to learn novel verbs in young Mandarin speakers

**DOI:** 10.1002/pchj.710

**Published:** 2023-12-17

**Authors:** Zhigang Li, Chenyue Liang, Jianing Zhong, Shuang Chen

**Affiliations:** ^1^ School of Psychology Zhejiang Normal University Jinhua China; ^2^ Intelligent Laboratory of Child and Adolescent Mental Health and Crisis Intervention of Zhejiang Province, Zhejiang Normal University Jinhua China

**Keywords:** function words, Mandarin, syntactic bootstrapping, verb learning

## Abstract

The present research examined whether Mandarin‐speaking children could use function words to learn novel verbs and recognize verbs in a new sentential context. In Experiment 1, 3‐ to 6‐year‐old children were taught two novel verbs supported by the verb marker “*zài*.” The 5‐ and 6‐year‐old children successfully used the function word “*zài*” to learn novel verbs, but the 3‐ and 4‐year‐olds failed to interpret the novel words as verbs. In Experiment 2 and 3, the children had to recognize the newly learned verbs in new sentences containing a different function word (a different verb‐biased marker “*le*” or a non‐verb‐biased marker “*shì*”). Results showed that the 5‐year‐old children could recognize the newly learned verbs with another verb‐biased marker “*le*,” but only the 6‐year‐old children could recognize the newly learned verbs with the non‐verb‐biased marker “*shì*.” The study verified that Mandarin‐speaking children could use the function word “*zài*” to determine a novel word as a verb and revealed that such an ability appeared between the ages of 4 and 5 years. In addition, the ability to extend a newly learned verb across different morphosyntactic markers is developed in 5‐ to 6‐year‐olds.

## INTRODUCTION

Our understanding of lexical development heavily relies on understanding the way children acquire verbs. Before acquiring the meaning of verbs, children must first determine a new word as a verb. The syntactic and morphological cues with which a new word is presented may play an important role. For example, in English, a new word is often a verb if it carries a morphological inflection—“ing.” Children may use the syntactic cues with which a new word appears to help discover its form class—a process called “syntactic bootstrapping” (Gleitman, 1990). However, in Mandarin Chinese, there is no inflectional morphology to tense or gender of verbs (Klein et al., 2000). Therefore, it is crucial to investigate whether syntactic bootstrapping can be applied in languages like Chinese.

Naigles (1990) first provided experimental validation of syntactic bootstrapping in verb learning. In the experiment, a group of 2‐year‐old children observed two events simultaneously—a duck performing an action on a bunny; a duck and a bunny doing the same action. During this experiment, they heard a sentence with a novel verb, such as “The duck is gorping the bunny” or “The duck and the bunny are gorping.” Based on the sentence frames, the children were able to select the correct event. The results strongly supported the idea that young children can use syntax to limit the meanings of novel verbs. Since then, there has been a significant amount of research exploring this ongoing issue (de Carvalho et al., 2019, 2021; Gertner et al., 2006; He & Lidz, 2017; Imai et al., 2005a; Kersten & Smith, 2002; Yuan & Fisher, 2006, 2009; Yuan et al., 2012). Support for the hypothesis of syntactic bootstrapping in verb learning has been demonstrated across various languages and age ranges. For example, English‐speaking infants as young as 18 months can use morphosyntactic information (“It's doking”) to learn novel verb meanings (He & Lidz, 2017). For English and French toddlers, morphological markers, inflections, and word order can serve as tools to limit the scope of meaning for new verbs. For instance, 28‐month‐old infants could infer the semantic content of the novel verb simply from hearing it in transitive or intransitive sentence frames without a concurrent scene (Yuan & Fisher, 2006, 2009). Additionally, 21‐month‐old infants can use word order to interpret sentences containing novel verbs (Gertner et al., 2006).

However, Mandarin is not rich in inflectional morphology (e.g., “‐ing/‐s”) and sentence meanings are sometimes not sensitive to changes in word order. For example, in Mandarin, to express the event that someone has finished a meal, sentences with different word orders can be used, such as “*chī* (eat) *guò* (complement) *fàn* (meal) *le* (verb marker)” or “*fàn chī guò le*.” Furthermore, Chinese is a language that allows argument dropping. For example, omitting the argument “*fàn* (meal)” was common when expressing the event that someone has finished a meal “*wǒ* (I) *chī* (eat) *guò* (complement) *le* (verb marker).” This casts doubt on young Mandarin learners' ability to use syntactic bootstrapping as a tool for verb learning.

Zhou et al. (2014) found that 3‐, 4‐, and 5‐year‐old Mandarin children and adults were able to use function words “了/*le*” and “着/*zhe*” to recognize events during online sentence comprehension. In addition, a recent corpus study of Mandarin child‐directed speech (CDS) demonstrated that function words were reliable syntactic markers for word form classes (Ma et al., 2019). Function words, such as “了/*le*,” “不/*bù*,” “没/*méi*,” “就/*jiù*,” “还/*hái*,” and “在/*zài*” are verb markers in CDS as these words have reliably higher co‐occurrence with verbs than nouns. Therefore, Mandarin‐speaking children may use these function words in verb learning.

### Young Mandarin‐speakers learn verbs using function words

Imai et al. (2008) examined the ability of 3‐ and 5‐year‐old Mandarin‐speaking children to learn novel verbs using the function word “*zài*” and the noun phrase “*yī* (one) *gè* (classifier) *dōng xī* (thing).” Each trial consisted of a standard event and a pair of test events. In the standard event, an actor performed a novel action on a novel object while saying, *“Kàn* (Look)*! Āyí* (Aunt) *zài* (verb marker) *X yī gè dōng xī* (one thing)!” During testing, a pair of test events was simultaneously shown side‐by‐side on the screen, indicating that the actor performed a novel action on the same object as the standard event (object‐same [OS] event) or performed the same action on a new object as the standard event (action‐same [AS] event). At the same time, the experimenter asked the children: “*Nǎ zhāng tú lǐ* (In which picture) *āyí* (aunt) *zài* (verb marker) *X yī gè dōng xī* (one thing)?” The results demonstrated that 5‐year‐old children could use the verb marker “*zài*” with aspect‐marking auxiliaries and the post‐verbal noun phrase to learn verbs when the researchers made the action perceptually more salient by removing the brief object holding segment of the video, but 3‐year‐old children made AS selection at chance level. Therefore, Chinese children may need to combine multiple cues to determine verbs as the presence of “*zài*” may not be a strong enough cue for young Chinese children (Imai et al., 2008). Furthermore, using the Beijing corpus of CHILDES, Lee and Naigles (2005) revealed that the post‐verbal noun phrase was a reliable cue for identifying transitive verbs. Thus, to examine the key role of the function word “*zài*” in verb learning, Ma et al. (2020) employed the same paradigm as Imai et al. (2008) to investigate the ability of 3‐ and 5‐year‐old Mandarin children to learn verbs only using the function word “*zài*” without the post‐verbal noun phrase “*Āyí* (Aunt) *zài* (verb marker) *X*.” The results showed that 5‐year‐old children could use the function word “*zài*” to learn verbs without aspect‐marking auxiliaries and verb arguments. Therefore, 5‐year‐old Mandarin children can use the function word “*zài*” as a relative strong cue for verb learning when the action was perceptually salient.

To verify whether Mandarin‐speaking children can use the pure function word “*zài*” to learn novel verbs, we used the sentence “*Kàn* (Look)! *Jiě‐jie* (Sister) *zài* (verb marker) *X*,” which is similar to that used in Ma et al. (2020). We dropped the verb argument to eliminate the cue of the post‐verbal noun phrase. Moreover, in the testing trial of the paradigm, the AS and OS events were presented simultaneously. If we used the transitive sentence “*Jiě‐jie* (Sister) *zài* (verb marker) *X yī gè wán jù* (a toy),” the OS event could be excluded by the noun phrase (*yī gè wán jù*/ a toy) as a noun could not be followed by an argument. Therefore, if participants selected the AS event in the test, we could not distinguish the role of the post‐verbal noun phrase from the role of the function word “*zài*” on the inference of verb meaning. In addition, the omission of verb arguments is common in Chinese, especially for child‐directed speech (Tardif, 1996). Hence, it is natural to drop the objects when they can be inferred from context. Furthermore, the instruction in Ma et al. (2020) was also “*Āyí* (Aunt) *zài* (verb marker) *X*,” which showed that 5‐year‐old Mandarin children can learn verbs. Therefore, the omission of object noun might not hinder toddlers' verb learning. Thus, we used the intransitive frame to examine the role of function words in verb learning.

Although previous research has indicated that Mandarin‐speaking children developed the ability to learn verbs using the function word “*zài*” from the ages of 3 to 5 years (Imai et al., 2008; Ma et al., 2020), the specific development during this stage remains unclear. Imai et al. (2005b) found that 4‐year‐old Japanese children could successfully extend verbs to different familiar objects, but 3‐year‐old children did not have this ability. In a subsequent study, Haryu et al. (2011) found that 4‐year‐old Japanese children could extend verbs to similar objects, but 3‐year‐old children still could not extend verbs successfully. The results demonstrated that 4‐year‐old children could extend verbs to familiar and similar objects, but this ability was not reflected in 3‐year‐old children, indicating that the development of some specific ability to learn verbs might also occur in the age from 3 to 4 years.

Furthermore, the ability of 5‐year‐old Mandarin‐speaking children to learn verbs is still immature (Imai et al., 2008; Ma et al., 2020). Imai et al. (2008) studied the development of verb learning in 3‐, 5‐, 6‐, and 8‐year‐old Mandarin‐speaking children. Due to the distraction caused by the object‐holding segment before learning, only 8‐year‐olds were able to successfully learn verbs in Imai et al. (2008). However, 5‐year‐olds were able to learn verbs after researchers removed this object‐holding segment to highlight the action, indicating that Chinese children aged 5 to 8 years might be susceptible to extra‐linguistic factors to different extent when learning new verbs. In addition, the study also examined the verb learning ability of adults and found that adults selected the AS scene 100% of the time. However, Ma et al. (2020) used the same paradigm and found that 5‐year‐olds selected the AS scene only 72% of the time. These results demonstrated that there may be potential for further development after the age of 5 years. However, the development of children after the age of 5 is under‐researched. Therefore, this study aimed to further explore and verify the development of verb learning supported by function words in 3–6‐year‐olds.

### Extension of newly learned verbs

Besides verb‐learning ability per se, previous studies also focused on the extension of newly learned verbs and found that young children could recognize newly learned verbs in novel situations across different event participants (e.g., extending to new actors or new objects) (Forbes & Poulin‐Dubois, 1997; Imai et al., 2005a; Imai et al., 2008; Maguire et al., 2002; Maguire et al., 2008; Naigles et al., 2005). For example, children as young as 31 months can extend verbs to different actors (Maguire et al., 2008), and 5‐year‐olds can extend verbs to a different object (Imai et al., 2005a). In order to learn a new verb, toddlers must not only extend it to new events, but also to new syntactic cues, which is necessary for children to attain adult‐like verb use (Theakston et al., 2002).

The extensions enable toddlers to interpret and use newly learned verbs flexibly in daily conversations. However, the ability to extend a novel verb to new syntactic cues received less attention than the extension to new actors or objects. An early paper (Naigles et al., 2005) explored the ability of 22‐month‐old children to recognize newly learned verbs in different argument structures. Children were taught novel verbs in a transitive sentence frame (“She's lorping the ball”). Afterward, this novel verb was heard in three different test frames: a transitive frame (“She's lorping the ball”), an intransitive frame (“She's lorping”), and a neutral frame (“Lorping”). The results demonstrated that children could correctly recognize verbs in both transitive and intransitive sentence frames, but failed to recognize verbs in neutral sentence frames. These results suggested that toddlers might need extra bootstraps to interpret newly learned verbs during the testing. As the neutral frames did not provide enough cues, they failed the test. Kline and Demuth (2014) also examined whether children aged 2 to 6 years could produce appropriate syntactic generalizations with novel verbs. They found that more than half of the toddlers (9 out of 16) could produce patient intransitive sentence with newly learned verbs, which were learned with transitive frame or vice versa. These two studies examined very young toddlers' extension of new verbs across different argument structure, showing that the testing frames mattered. Young English speakers could extend new verbs across transitive and intransitive sentences, but encountered difficulties without the help of argument structure during the test.

Besides adapting to different argument structure, verbs could be associated with different morphosyntactic markers, e.g., “lorping vs. lorped”. Toddlers might acquire a verb and its inflections at different stages. For example, Theakston et al. (2002) examined the acquisition of the different forms of the verb “Go” based on data from 11 children followed longitudinally between the ages of 2 and 3 years. They illustrated the order of acquisition of different forms of “Go” as the “Going,” “Goes,” and “Gone” was produced later than “Go.” A subsequent study confirmed that the different inflectional forms of the same verb were affected by the input frequency (Tatsumi et al., 2018). Therefore, it is necessary to explore the extension of newly learned verbs across different morphosyntactic markers, yet this ability per se, as well as its development, remains under‐studied. As young Mandarin speakers may use function words to learn novel verbs (Ma et al., 2020), it is necessary to explore the extension of verbs learned under one class marker (e.g., “*zài*”) to a different morphosyntactic markers (e.g., “*le*”). Therefore, the present study also aimed to further investigate whether Mandarin‐speaking children could interpret newly learned verbs with different morphosyntactic markers and to investigate the development of this ability.

In Mandarin, the function word “*zài*” is usually used to indicate the progressive aspect of verbs, and the frequency of co‐occurrence with the verb is 40% (Ma et al., 2019). The function word “*le*” is usually used to indicate the perfective aspect of verbs, and the frequency of co‐occurrence with the verb is 46% (Ma et al., 2019). Furthermore, the function word “是/*shì*” can precede verbs, nouns, and adjectives (Wang, 2019), so it is not a reliable marker to identify the form class of a new word. Therefore, we used the function word “*zài*” as a verb marker in a standard event, and different function words, including a different verb maker “*le*” with higher frequency of co‐occurrence with the verb (Experiment 2) and the non‐verb‐biased marker “*shì*” (Experiment 3) in test events. By using markers in testing questions different from those in learning events, we explored whether Mandarin‐speaking children could interpret newly learned verbs with different morphosyntactic markers and the development of this ability.

### The present study

In general, this study used three experiments to investigate the development of verb learning supported by function words and the ability of extending newly learned verbs across different morphosyntactic markers in 3–6‐year‐olds using the same paradigm as Ma et al. (2020). Experiment 1 explored the development of verb learning supported by the function word “*zài*” in 3–6‐years‐olds. Experiments 2 and 3 aimed to explore whether children could recognize newly learned verbs in new sentences with a different verb‐biased marker (“了/*le*”) or a non‐verb‐biased marker (“是/*shì*”). See Table 1 for instruction examples used in the three experiments.

**TABLE 1:** Instructions for Experiments 1–3.

Experiment	Instructions for learning	Questions for test
Exp. 1	“*Kàn* (Look)! *Jiě‐jie* (sister) *zài* (marker) *X*.” “Look! Sister is X‐ing.”	“*Nǎ biān* (Which side) *zài* (marker) X?” “Which side is X‐ing?”
Exp. 2		“*Nǎ biān* (Which side) *X le* (marker)?” “Which side is X‐ed?”
Exp. 3		“*Nǎ biān* (Which side) *shì* (marker) X?” “Which side is X?”

## EXPERIMENT 1

### Participants

A total of 224 children were recruited as participants from several preschools in Zhejiang, a province in East China. The participants were 51 3‐year‐old children (*M* = 43.3 months, *SD* = 2.9 months, range = 36.7–47.9 months; 23 girls), 61 4‐year‐old children (*M* = 54.1 months, *SD* = 3.7 months, range = 48.1–59.9 months; 29 girls), 66 5‐year‐old children (*M* = 65.9 months, *SD* = 3.3 months, range = 60.3–71.6 months; 34 girls), and 46 6‐year‐old children (*M* = 75.0 months, *SD* = 1.6 months, range = 72.2–77.5 months; 21 girls). Four children were excluded due to experimenter errors. The children were included once their parents or legal guardians gave oral consent to participate in the study. Each child only attended to one experiment of the study, instead of repeatedly attending to all experiments. This study was approved by the Research Ethics Committee of Zhejiang Normal University.

### Materials and procedure

The children were tested individually in a quiet room at a preschool. The visual stimuli were presented on a 14‐inch laptop computer, whereas the auditory stimuli were presented by the experimenter, who is a native speaker of Mandarin. The stimulus presentation method was identical to that used in other studies (Imai et al., 2005a; Imai et al., 2008; Ma et al., 2019). Before the test trials, the children received two warm‐up trials to ensure that they could understand the procedure and point to one of two test video clips.

The two warm‐up trials were presented to all the children in a fixed order. In the first trial, two familiar animals (cats and dogs) were presented to the children simultaneously. The experimenter asked the children: “*Nǎ biān* (which side) *shì* (is) *xiǎo māo* (cat)?” The second trial consisted of a standard event and a pair of test events. In the standard event, an actor moved a teddy bear up and down while saying, “*Kàn* (Look)! *Jiě‐jie* (Sister) *zài* (marker) *X*.” During the test, a pair of events was simultaneously shown side‐by‐side of the screen: the actor moved a teddy bear up and down (exactly the same) or pulled and stretched a toy koala (completely different). At the same time, they asked the children, “*Nǎ biān* (Which side) *shì* (is) *X*?”

**FIGURE 1:**
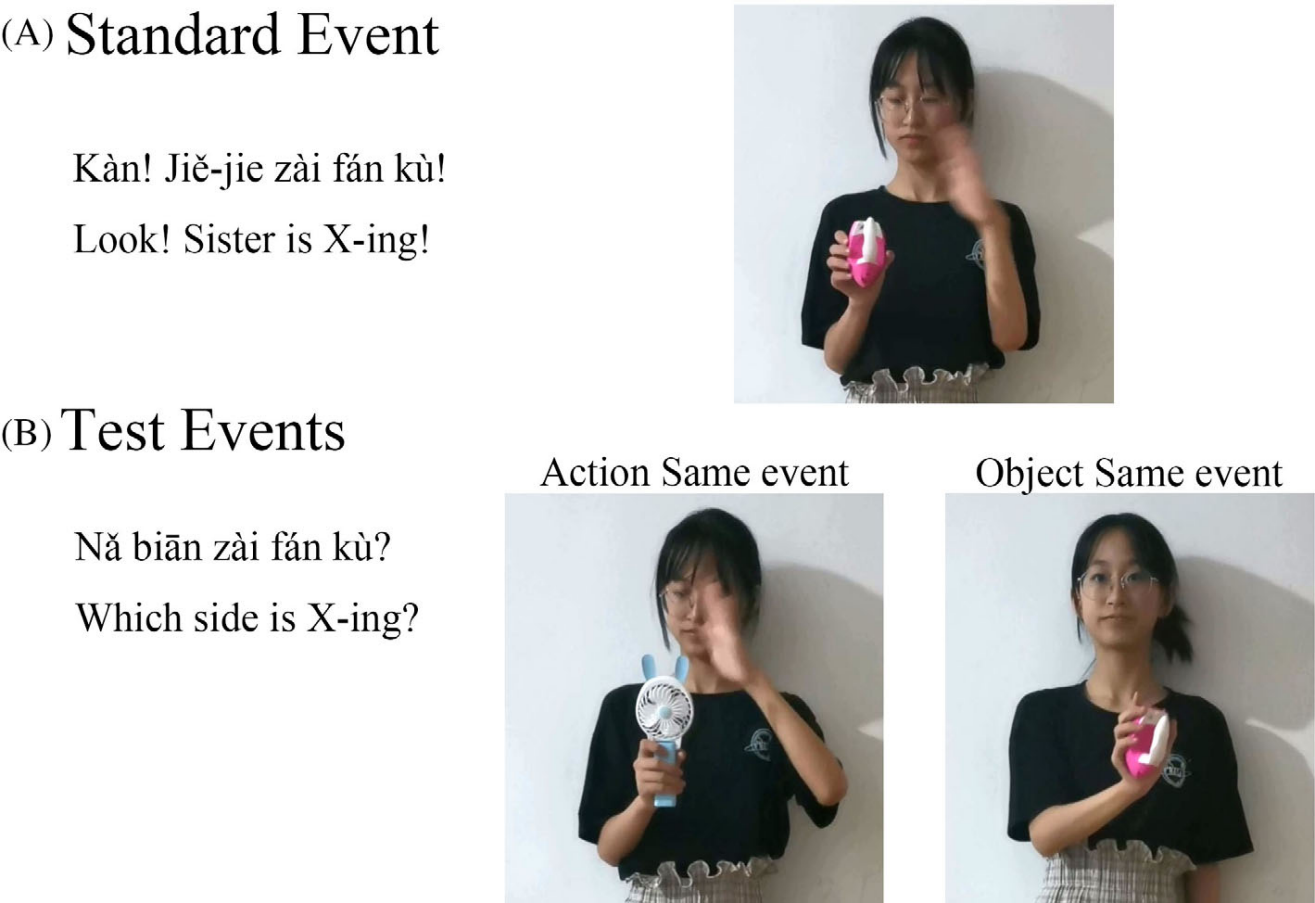
An example trial in Experiment 1.

After the warm‐up trials, the children underwent two test trials. The order of the two test trials was counterbalanced between the children. In each trial, the children needed to learn new words by watching videos. The new words were meaningless (*fán kù, wàn nǎn*) and composed of two Chinese characters. Each trial was composed of a standard event and a pair of test events. In the standard event, an actor performed a novel action on a novel object while saying: “*Kàn* (Look)! *Jiě‐jie* (Sister) *zài* (marker) *X*.” In the test event, a pair of events was simultaneously shown side‐by‐side on the screen, indicating that the actor completed a novel action on the same object as the standard event (OS event) or completed the same action on a new object as the standard event (AS event). At the same time, the children were asked: “*Nǎ biān* (Which side) *zài* (marker) *X*?” The children were asked to point out the corresponding video using their hands. If the child chose the AS scene, this was regarded as a correct response. All the videos were created using the same actor. Each video was presented for 20 s, and the sound stimulation was repeated twice. Table 2 and Figure 1 illustrate the stimuli and design of the two test trials.

**TABLE 2:** A summary of stimuli for the two test trials in Experiment 1.

	Standard event		Object‐same test		Action‐same test
Trial 1	A woman is patting the object with her left hand.	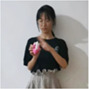	A woman is holding the object in her right hand while moving it in a circular motion.	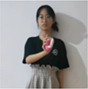	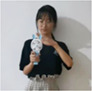
Trial 2	A woman is holding the object with her right hand while moving it from side to side.	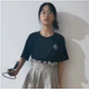	A woman is lifting and lowering the object using both hands.	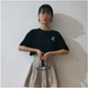	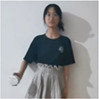

### Results

**FIGURE 2:**
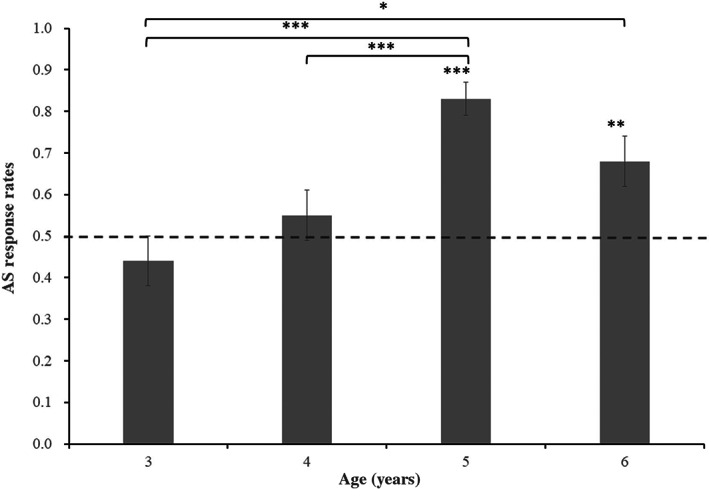
The 3–6‐year‐olds' action‐same (AS) response rates in Experiment 1 (error bars reflect *SEM*). *p < .05. **p < .01. ***p < .001.

Separate one‐sample *t*‐tests were used to compare the AS response rates in each age group against chance (0.5). The results demonstrated that 3‐year‐olds (*M* = 0.44, *SD* = 0.43; *t*(50) = −0.97, *p* = .335) and 4‐year‐olds (*M* = 0.55, SD = 0.46; *t*(60) = 0.83, *p* = .410) did not differ significantly from chance, whereas 5‐year‐olds (*M* = 0.83, SD = 0.35; *t*(65) = 7.64, *p* < .001) and 6‐year‐olds (*M* = 0.68, SD = 0.43; *t*(45) = 2.94, *p* = .005) were significantly above chance. The experimental results are illustrated in Figure 2. To examine whether the pattern of results holds for the distribution of individuals, the number of children who made correct choices in both trials was calculated. The sign test results indicated that the number of 3‐year‐olds was significantly lower than chance (16 of 51, *p* = .011), the numbers of 4‐year‐olds (29 of 61, *p* = .798) and 6‐year‐olds (28 of 44, *p* = .096) were not significantly different from chance, and the number of 5‐year‐olds (53 of 66, *p* < .001) was significantly above chance.

A logistic mixed effect analysis was used to test the difference in verb learning between different age groups, which included data from 448 trials (2 trials × 224 subjects). The participants and items were entered into the model as random intercepts; further, age group served as a fixed‐effects variable, and the responses for each trial were used as a dependent variable. The results found a significant age group effect: 5‐year‐old (*Z* = 3.50, *p* < .001) and 6‐year‐old (*Z* = 2.26, *p* = .024) children's AS response rates were significantly higher than that of 3‐year‐old children (*Z* = 3.61, *p* < .001). In addition, 5‐year‐old (*Z* = 3.61, *p* < .001) and 6‐year‐old (*Z* = 1.86, *p* = .063) children's AS response rates were higher than that of 4‐year‐old children. Besides, the difference between the performance of 3‐year‐old children and that of 4‐year‐old children was not significant (*Z* = 1.29, *p* = .196). The difference between the performance of 5‐year‐old children and that of 6‐year‐old children was also not significant (*Z* = −1.58, *p* = .115).

### Discussion

This experiment found that 5‐ and 6‐year‐old Mandarin‐speaking children could use the function word “*zài*” to learn verbs. However, 3‐ and 4‐year‐old children did not have this ability. This result is consistent with Ma et al. (2020), indicating that 5‐year‐old Mandarin speakers can use function words to distinguish nouns and verbs but not 3‐year‐olds. Moreover, we revealed a significant difference between 4‐ and 5‐year‐old children's performance as well as equivalent performance for 3‐ and 4‐year‐old children, indicating that the ability to use function words to learn verbs appears between the ages of 4 and 5 years.

We found that 3‐year‐olds selected the AS events at the chance level. One possibility was that they did not have a robust understanding of the function of verb marker “*zài*” (Ma et al., 2020) or they had difficulties using it as a cue to recognize a new word as a verb. As a couple of studies have shown that Mandarin leaners aged 30–31 months have mastered the imperfective meaning of “*zài*” when it appears before familiar verbs (Su & Naigles, 2021; Yang et al., 2018), 3‐year‐old Mandarin learners may already have a robust understanding of “*zài*.” Therefore, they might just have difficulties using “*zài*” as a cue to recognize a new verb.

Furthermore, 3‐year‐olds might also have difficulty extracting the common action between the standard event and AS event (Haryu et al., 2011) or mapping the novel word to the whole event (Imai et al., 2005a). Therefore, their performances were instable across studies. For example, some previous studies also found that 3‐year‐olds selected the AS events at chance level in similar experimental conditions (Imai et al., 2005a; Ma et al., 2020), whereas 3‐year‐olds showed below‐chance performance in other studies (Haryu et al., 2011; Imai et al., 2005b) using the same paradigm. The unstable performance might result from the fact that 3‐year‐olds' performances were largely influenced by stimuli and learning contexts. For instance, in Imai et al. (2005b), familiarity of the objects used in the AS events affected 3‐year‐old Japanese toddlers' performance showing chance‐level performance for familiar objects but below‐chance level for unfamiliar objects, whereas dropping argument did not affect their performance. However, there were mixed results from different studies. Haryu et al. (2011) found that 3‐year‐old Japanese toddlers selected the AS test event only 25.0% of the time when the object was dissimilar between the AS event and the standard event, whereas in Imai et al. (2005a), 3‐year‐old Japanese toddlers selected the AS event 64% of the time using dissimilar objects. Therefore, 3‐year‐olds may not readily learn novel verbs relying on one cue.

Only limited studies have examined the verb learning of children at age 4 years old using a similar paradigm (Haryu et al., 2011; Imai et al., 2005b). These studies found that 4‐year‐olds selected the AS event at the chance level, which is consistent with our results. However, compared to 3‐year‐olds, familiar objects or similar objects can bootstrap verb learning and extension for 4‐year‐old children (Haryu et al., 2011; Imai et al., 2005b). Therefore, there may be a potential for development between the ages of 3 and 4 years, which may not get expressed in this paradigm using novel and dissimilar objects. Furthermore, the more substantial development occurs between the ages of 4 and 5 years, as revealed by the present experiment with the superior performance for 5‐year‐olds. A previous study (Wakefield et al., 2018) also found that children between the ages of 4.5 and 5.5 years could generalize the novel verb to an action on a different object, and that gesture about the action could improve the generalization. Therefore, developing the ability to extract action from the whole event and generalizing the action to a new object may be the key factor underlying the performance improvement of 4‐ to 5‐year‐olds.

Although 5‐year‐old Mandarin‐speaking children can use the function word “*zài*” to learn verbs, their ability to learn verbs is still immature. Additionally, previous studies have found that children tend to be conservative in recognizing verbs in novel situations and sentences (Forbes & Poulin‐Dubois, 1997; Imai et al., 2005a, 2008; Maguire et al., 2002; Maguire et al., 2008; Naigles et al., 2005). This implies that children do not have a robust understanding of newly learned verbs. It is unclear whether young Mandarin‐speaking children can recognize verbs with a different verb‐biased marker. In Mandarin Chinese, there are four primary grammatical aspect markers for verbs, including the progressive morpheme “*zài*,” as well as the perfective morpheme “*le*,” which emerges earliest among 1‐2‐year‐old children (Su & Naigles, 2021). A previous study revealed that 30‐month‐old Mandarin‐speaking children were sensitive to the aspects of events involving the marker “*le*” (Yang et al., 2018). These results indicated that Mandarin‐speaking children have learned and can interpret the function word “*le*” early in language development. Therefore, we used the function word “*le*” in a testing sentence to explore whether, after learning the verbs under the verb‐marker “*zài*” (“*Kàn*! *Jiě‐jie zài X*”/“Look! Sister is X‐ing”), 5‐year‐olds could recognize the verbs with a different verb‐biased marker “*le*” (“*Nǎ biān X le*?”/“Which side is X‐ed?”).

## EXPERIMENT 2

### Participants

The participants in Experiment 2 were 39 5‐year‐old children (*M* = 65.5 months, *SD* = 3.0 months, range = 60.6–70.7 months; 22 girls) from a preschool in Zhejiang. If the parents or legal guardians gave their oral consent to participate, the children were included. This study was approved by the Research Ethics Committee of Zhejiang Normal University.

### Materials and procedure

The experimental materials and procedures were similar to those used in Experiment 1, but four test trials were created (see Table 3). The warm‐up trials were the same as Experiment 1. For the experiment trials, the standard event and instruction (i.e., “*Kàn* (Look)! *Jiě‐jie* (Sister) *zài* (marker) *X*") was the same as Experiment 1; however, the questions in the test phase were modified. Instead of providing the verb‐marker “*zài*,” we questioned the children using a new sentence with the new verb‐marker “*le*”: “*Nǎ biān X le*? (Which side is X‐ed?)”

**TABLE 3:** A summary of events for the four test trials in Experiment 3.

	Standard event		Object‐same test		Action‐same test
Trial 1	A woman is holding the object in the palm of her left hand while moving in a circular motion.	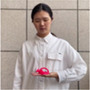	A woman is balancing a red novel toy on the right index finger while spinning it horizontally.	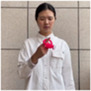	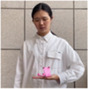
Trial 2	A woman is holding the object in her right hand while patting her left shoulder with it.	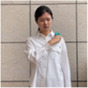	A woman is tossing a blue novel toy up and down with both hands.	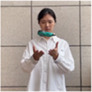	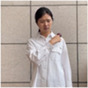
Trial3	A woman is holding the object with her right hand while lifting and lowering it diagonally.	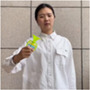	A woman is tossing a blue novel toy in the air using one hand and catching it with the other hand.	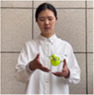	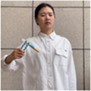
Trial 4	A woman is dangling the object with her left wrist.	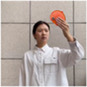	A woman is flipping an orange novel toy using both hands in front of the chest.	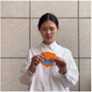	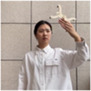

### Results

A separate one‐sample *t*‐test found that the performance of 5‐year‐olds (*M* = 0.85, *SD* = 0.32; *t*(38) = 6.8, *p* < .001) was significantly above chance. To examine whether the pattern of results holds for the distribution of individuals, the number of children who made correct choices in both trials was counted. The sign test results demonstrated that the number of 5‐year‐olds with correct choices (32 of 39, *p* < .001) was significantly higher than chance.

### Discussion

Experiment 2 revealed that 5‐year‐old Mandarin‐speaking children could recognize newly learned verbs with the new verb‐marker “*le*.” In Experiment 1, 5‐year‐olds could generalize the newly learned verb to a different object when the verb was preceded with the same verb marker. On top of this, Experiment 2 revealed that they could also generalize the newly learned verb to a different verb‐biased marker. Taken together, these two experiments gave clear and strong evidence that the 5‐year‐olds were able to make use of function words when learning and generalizing new verbs.

Researchers may be concerned that “*zài*” and “*le*” indicate different aspects. The function word “*zài*” expresses a “progressive” aspect mainly to indicate an action or event in progress, whereas “*le*” characterizes the situation as “perfective.” However, the differences do not necessarily imply the two function words cannot be applied to the same event, as they can be used to describe the same situation/event in terms of which part of the sentence's descriptive content is asserted (Klein et al., 2000). For example, previous studies usually used the different parts of the same events to test the children's comprehension of aspect markers “*zài*” and “*le*” (Chen et al., 2022). As the events used in the experiments were durable and came to a conclusion as well, the different markers could characterize the progress (“*zài*”) or its entirety (“*le*”), respectively. Therefore, it is plausible to apply “*zài*” and “*le*” in different sentences to the same event. Nevertheless, the experiment could not tell whether the marker “*le*” in the test questions changed the interpretation of the aspectual properties of the novel verb. Although previous studies (e.g., Chen et al., 2022; Li & Bowerman, 1998) provided evidence that young Mandarin children interpreted different aspectual properties of known verbs according to the verb‐marking function words, the present experiment did not construct events reflecting different aspects to test such ability for newly learned verbs. However, this can be an empirical issue that requires future investigation.

The function words in Chinese may co‐occur with content words from different grammatical categories. For instance, although the verb‐biased marker “*zài*” has a greater tendency to co‐occur with verbs rather than nouns, it can come before nouns (e.g., *zài jiā*, at home) or prepositions (e.g., *zài gēn tā chī fàn*, have a meal with her). Therefore, “*zài*” is not a foolproof indicator for a new verb. Moreover, in real life, children may encounter a new verb preceded by “*zài*” for the first time, but encounter a different function word for the second time. Experiment 2 has indicated that if the different function word is a verb‐marker, such as “*le*,” 5‐year‐olds could recognize the verb successfully. However, it may be challenging for young Chinese learners to recognize when the new verb is preceded by a non‐verb‐biased marker, like “*shì*,” which often occurs in daily life learning situations. Therefore, Experiment 3 aimed to investigate whether 5‐ and 6‐year‐olds could maintain their interpretation of a new verb even when it was preceded by the marker “*shì*” in the testing phase and further explore whether 3‐ and 4‐year‐olds would be misled by the marker “*shì*.”

## EXPERIMENT 3

### Participants

For Experiment 3, a total of 244 children were recruited as participants from several preschools in Zhejiang. The participants were 51 3‐year‐old children (*M* = 43.7 months, *SD* = 2.9 months, range = 36.4–47.9 months; 28 girls), 73 4‐year‐old children (*M* = 54.1 months, *SD* = 3.6 months, range = 48.0–59.9 months; 33 girls), 73 5‐year‐old children (*M* = 64.4 months, *SD* = 3.1 months, range = 60.2–71.8; 28 girls), and 47 6‐year‐old children (*M* = 75.3 months, *SD* = 1.7 months, range = 72.0–82.3 months; 17 girls). Two additional children were excluded because their birthday information was missing. If the parents or legal guardians gave their oral consent to participate in the study, the children were included. This study was approved by the Research Ethics Committee of Zhejiang Normal University.

### Materials and procedure

The experimental materials and procedures were similar to those of Experiment 1 and included two warm‐up trials and two test trials. The warm‐up trials were the same as in Experiment 1. However, during the test trial, the instruction in the test phase was modified: instead of providing the verb‐marker “*zài*,” the children were questioned using a new sentence with the non‐verb‐biased marker “*shì*”: “*Nǎ biān shì X?* (Which side is X?)”

### Results

**FIGURE 3:**
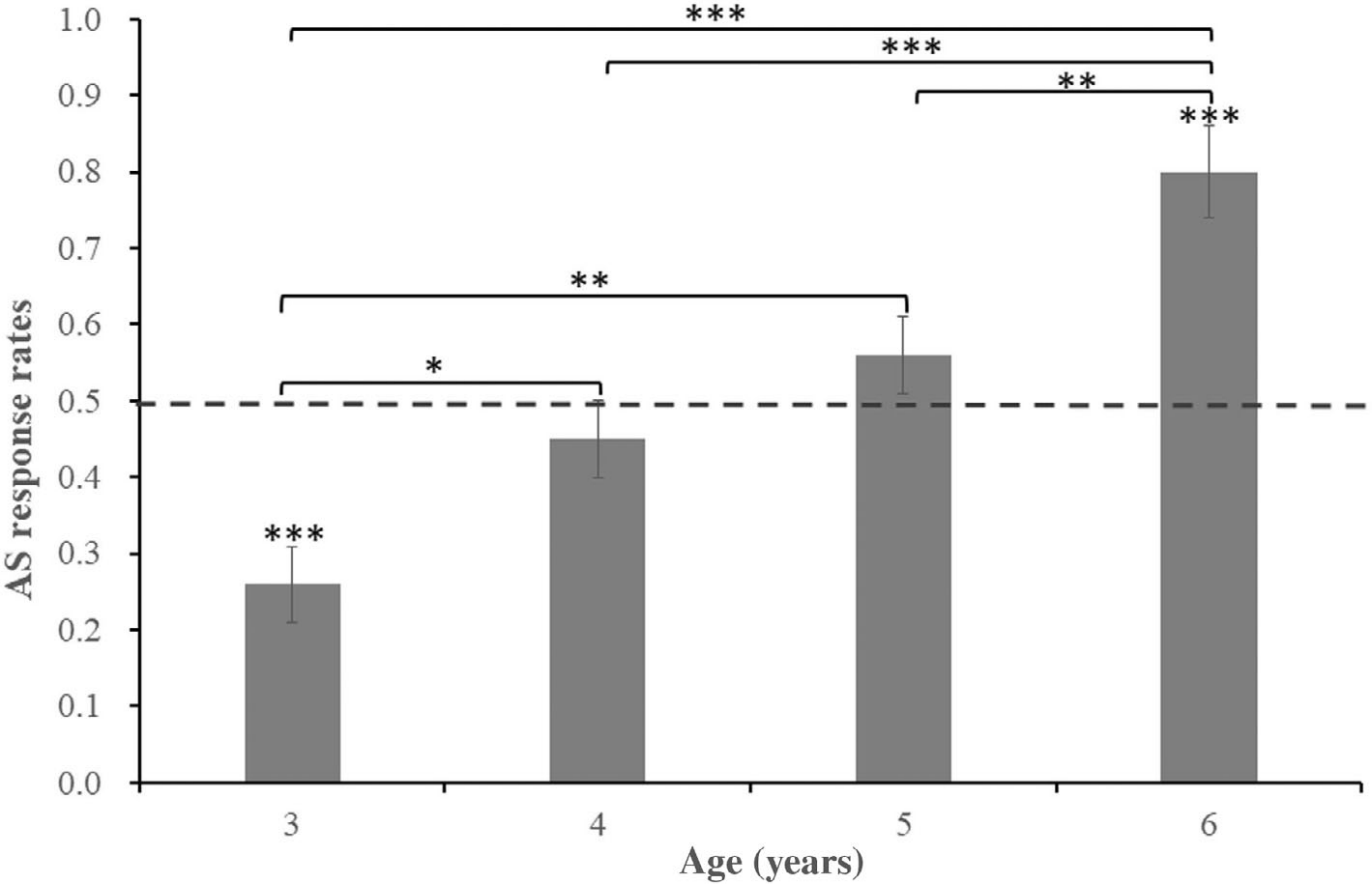
The 3–6‐year‐olds' action‐same (AS) response rates in Experiment 3 (error bars reflect *SEM*). *p < .05. **p < .01. ***p < .001.

Separate one‐sample *t*‐tests were used to compare the AS response rates in each age group against chance (0.5). The results found that 3‐year‐olds (*M* = 0.26, *SD* = 0.39; *t*(50) = −4.29, *p* < .001) were significantly lower than chance, 4‐year‐olds (*M* = 0.45, *SD* = 0.42; *t*(72) = −0.98, *p* = .330) and 5‐year‐olds (*M* = 0.56, *SD* = 0.46; *t*(72) = 1.14, *p* = .260) were not significantly different from chance, and 6‐year‐olds (*M* = 0.80, *SD* = 0.39; *t*(46) = 5.30, *p* < .001) were significantly above chance. The results are illustrated in Figure 3. To examine whether the pattern of results holds for the distribution of individuals, the number of children who made correct choices in both trials was calculated. The sign test results demonstrated that the number of 3‐year‐olds (9 of 51, *p* < .001) and 4‐year‐olds (22 of 73, *p* < .001) with correct choices was significantly lower than chance, the number of 5‐year‐olds (36 of 73, *p* = 1) was not significantly different from chance, and the number of 6‐year‐olds (36 of 47, *p* < .001) was significantly higher than chance.

A logistic mixed effect analysis was used to test the difference in verb learning between different age groups, which included data from 488 trials (2 trials × 244 subjects). The participants and items were entered into the model as random intercepts and age groups served as a fixed‐effects variable and the responses for each trial were used as a dependent variable. The results found a significant age group effect: 4‐year‐old (*Z* = 2.51, *p* = .012), 5‐year‐old (*Z* = 3.15, *p* = .001), and 6‐year‐old (*Z* = 4.34, *p* < .001) children's AS response rates were significantly higher than those of 3‐year‐old children; and 6‐year‐old children's AS response rates were significantly higher than those of 4‐year‐old (*Z* = 3.19, *p* < .001) and 5‐year‐old (*Z* = 3.27, *p* = .001) children. In addition, the difference between the AS response rate of 4‐year‐old children and that of the 5‐year‐old children was not significant (*Z* = 1.59, *p* = .112).

**FIGURE 4:**
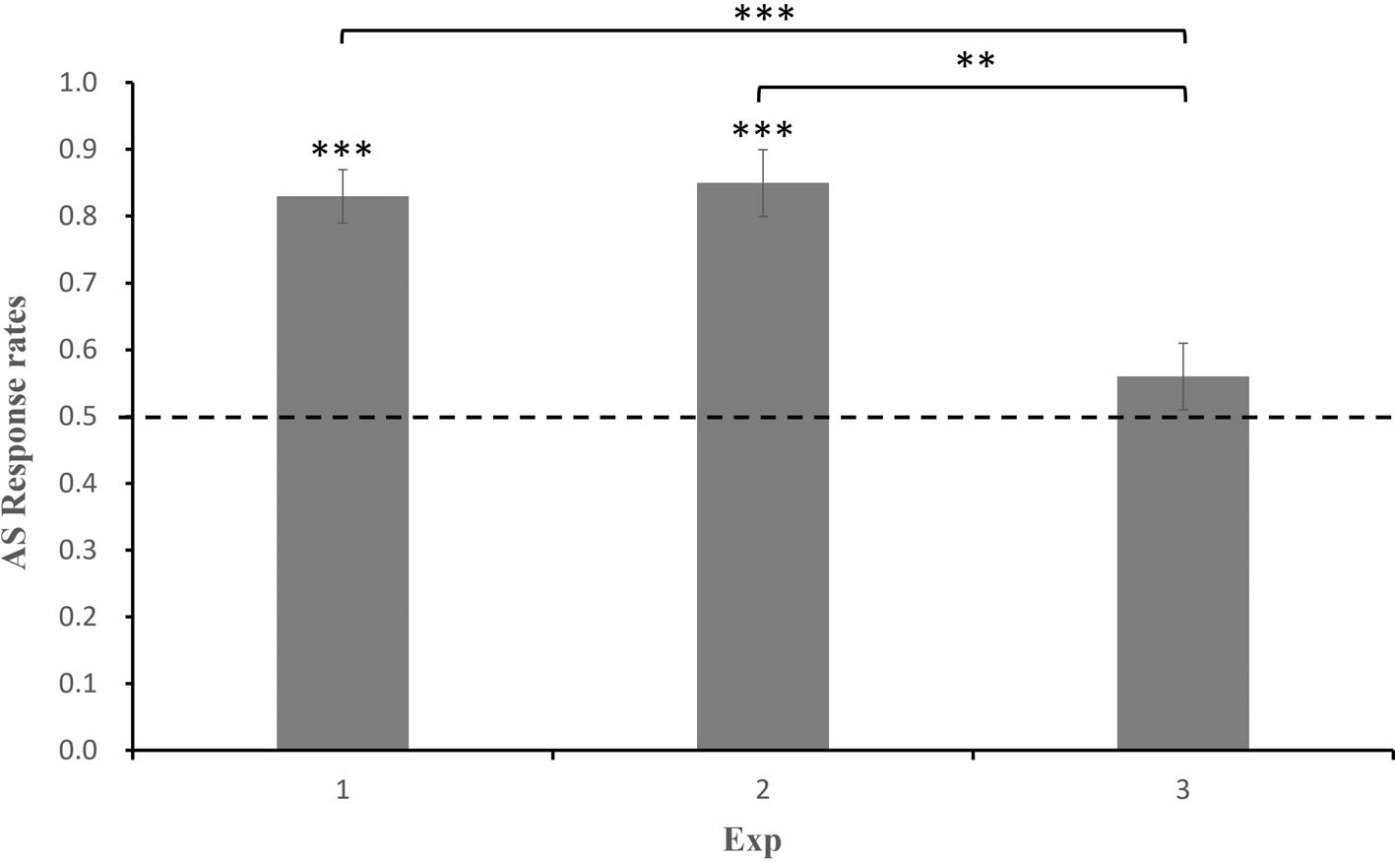
The 5‐year‐olds' action‐same (AS) response rates of Experiment 1–3 (error bars reflect *SEM*). **p < .01. ***p < .001.

A one‐way analysis of variance was performed on the AS response rates of 5‐year‐old children in the three experiments to compare the effects of different function word cues on 5‐year‐old children's verb learning. The results demonstrated that the main effect of the experiment was significant (*F*(2,175) = 10.5, *p* < .001). Multiple comparisons using Bonferroni correction revealed that the AS response rates of Experiment 1 (*p* < .001) and Experiment 2 (*p* = .001) were significantly higher than that in Experiment 3, and there was no significant difference between Experiment 1 (*p* = 1) and Experiment 2 (Figure 4).

### Discussion

Experiment 3 found that only 6‐year‐old Mandarin‐speaking children could recognize verbs with the non‐verb‐biased marker “*shì*,” whereas the 5‐year‐olds no longer generalized the newly learned verb to AS events. When the sentence used in the testing trial included a verb marker “*zài*” in Experiment 1 or a verb‐biased marker “*le*” in Experiment 2, 5‐year‐old children could successfully select the AS event, whereas their performance was at the chance level when the new word followed a non‐verb‐biased marker “*shì*” in Experiment 3. One possibility was that the 5‐year‐olds refused to generalize the verbs to a new context when there was no verb‐biased syntactic cue but a non‐biased marker. However, the 6‐year‐olds could better interpret or guess the goals of the experiment. Therefore, they maintained the initial verb interpretation. Another possibility was that 5‐year‐old children's representation of the novel verb was fragile, requiring cues during testing to enable them to generalize a newly learned verb to the same action. In study 3 of Imai et al. (2008), 5‐year‐old Mandarin‐speaking children learned bare novel words (e.g., “Look, X!”) and heard a sentence that included “*shì*” in the testing trial “*Nǎ* (which) *gè* (classifier) *tú* (picture) *zhǐ* (indicate) *de* (Nominal particle, auxiliary word) *shì* (is) *X?*” They found that 5‐year‐olds selected the AS event significantly below chance (0.22). Consistent with the syntactic role of “*shì*,” children could not use the word “*shì*” to bias verb meaning. Although 5‐year‐olds of the present study learned the novel verb and used the word “*zài*” in the standard event, their interpretation may be conservative and may need further bootstrapping during the test.

For 3‐year‐old Mandarin‐speaking children, the AS response rates were significantly lower than chance, indicating that they preferred to choose the OS scene, which differed from the performance of 3‐year‐olds in Experiment 1. Although in Mandarin Chinese, “*shì*” can precede verbs, nouns and adjectives (Wang, 2019), its prediction power for verbs and for nouns might differ. For instance, it may be usually followed by nouns in young children's language exposure as adults often point to an object by saying “*zhè shì X*/ This is X" to teach the child a novel noun. Since we failed to obtain the data of the co‐occurrence frequencies of “*shì*” with nouns vs. verbs from previous research, we calculated the co‐occurrence frequencies by ourselves using the corpus of modern Chinese of the State Language Commission of China (corpus.zhonghuayuwen.org), which provided part‐of‐speech tagging. According to the first 100 sentences containing “*shì*” from the corpus, the co‐occurrence frequency of “*shì*” with nouns (47%) was higher than with verbs (28%). Therefore, the word “*shì*” in the testing sentence may be a misleading cue that leads 3‐year‐olds to interpret the novel word as a noun. It may also be a confusing cue for 5‐year‐olds that interfered with their performance in this experiment.

## GENERAL DISCUSSION

This study examined the development of verb learning supported by function words in 3‐ to 6‐year‐old Mandarin‐speaking children. Experiment 1 demonstrated that 5‐ and 6‐year‐olds could use the function word “*zài*” to learn verbs, but 3‐ and 4‐year‐olds lack this ability. Experiment 2 revealed that 5‐year‐olds could generalize the newly learned verb to a different verb‐biased marker “*le*.” Thus, the study demonstrated that 5‐year‐old Mandarin‐speaking children could use the function word “*zài*” to learn verbs. More precisely, this ability appears between the ages of 4 and 5 years. Experiment 3 further showed that only 6‐year‐olds could recognize the newly learned verbs preceded by a non‐verb‐biased‐marker “*shì*.”

### Can young Mandarin‐speaking children use function words to learn verbs?

Using the Beijing corpora of CHILDES, Ma et al. (2019) found that function words were reliable morphosyntactic cues to distinguish between nouns and verbs in Mandarin child‐directed speech (CDS). There are four common function words used as verb aspect markers in Mandarin: perfective aspect marker “*le*,” progressive aspect marker “*zài*,” experiential perfective aspect marker “*guò*,” and imperfective aspect marker “*zhe*” (Chen & Shirai, 2010). Using the visual world eye‐tracking paradigm, a previous study (Zhou et al., 2014) found that 3‐year‐old Mandarin‐speaking children were able to use the temporal information encoded in the aspect markers “*le*” and “*zhe*” to recognize which event the verb referred to. Chen and Durrleman (2022) also found that 4‐ to 6‐year‐old typically developing Mandarin‐speaking children successfully recognized events corresponding to sentences containing aspect markers “*zài*,” “*zhe*,” and “*le*.” In addition, Yang et al. (2018) found that Mandarin‐speaking children as young as 30 months had a sensitivity to the distinction between the perfective marker “*le*” and the imperfective marker “*zhe*.” In sum, previous studies indicated that verb makers “*zài*,” “*zhe*,” and “*le*” helped young children's interpretation of familiar verb meanings.

Although previous studies have found that Mandarin children can use verb markers to recognize the temporal information of events (Ma et al., 2019; Ma et al., 2020; Yang et al., 2018; Zhou et al., 2014; Zhou & Ma, 2018), the specific role of function words in new verb learning received less attention. The present study, together with Imai et al. (2008) and Ma et al. (2020), showed that Mandarin‐speaking children's ability to identify the form class of a new word as a verb with the presence of “*zài*” in the instruction sentence emerged from the age of 5 years for Mandarin‐speaking children.

### The developmental of verb learning supported by function words

The present study explored and verified the development of verb learning via function words in 3‐ to 6‐year‐old Mandarin‐speaking children. The results indicated that 5‐year‐olds could interpret a new word as a verb according to function word “*zài*” and extend the newly learned verb to an event with a different object, but that 3‐year‐olds do not have this ability, which is consistent with the findings of previous studies (Ma et al., 2020). The results also confirmed that verb argument and aspect‐marking auxiliaries (e.g., “*zài*,” “*zhèng zài*,” and “*yī zhí zài*”) used in Study 5 of Imai et al. (2008) were not necessary cues for 5‐year‐old Chinese children to learn verbs. Furthermore, the performance of 4‐year‐olds also revealed that this ability may appear between 4 and 5 years of age. Previous studies found that 4‐year‐old Japanese children could learn novel verbs in a similar paradigm when the objects in the AS events were familiar or similar to objects in the standard events, but that 3‐year‐old children did not prefer the AS events even with the scaffolding of familiar or similar objects (Haryu et al., 2011; Imai et al., 2005b). Since the objects used in the present study were unfamiliar and dissimilar, we could not conclude whether the performance between 3‐ and 4‐year‐olds would differ with familiar or similar objects. Young children could make use of multiple cues to learn words and their learning was easily affected by stimuli and contexts. Therefore, it is possible that development of using cues other than the function word “*zài*” may occur from 4 to 5 years of age; however, this requires future investigation.

Although a previous study found that the function word “*zài*” had a higher co‐occurrence frequency with verbs (Ma et al., 2019) than other grammatical categories, it does not imply that the function word “*zài*” can only be used as a verb marker. More specifically, “*zài*” belongs to various parts of speech, including verbs, prepositions, and adverbs in Chinese (Gao, 2015). Thus, owing to the grammatical richness of the word “*zài*” in Chinese, it brings difficulty for young Mandarin‐speaking children to use “*zài*” to learn verbs. When the comprehension of “*zài*” increased steadily with children's age (Li & Bowerman, 1998), the marker “*zài*” could become a more reliable and robust cue. Before this, children need multiple cues besides this imperfect syntactic cue to learn new verbs.

### Does successful syntactic bootstrapping occur later in Mandarin‐speaking children?

Previous studies revealed successful syntactic bootstrapping in verb learning at an early age in some languages (i.e., 18‐month‐old French learners in de Carvalho et al., 2019; 18‐month‐old English learners in He & Lidz, 2017). The present study showed that Mandarin learners failed to use function words to identify verbs even at age 4 years. Successful syntactic bootstrapping seems to occur later among Chinese‐ speaking children than in English‐speaking children. Although cross‐linguistic studies using the same paradigm for English, Japanese, and Chinese learners showed similar development of verb acquisition, Chinese learners were more easily prone to interference with subtle contextual cues when learning novel verbs (Imai et al., 2008). One possible reason could be attributed to the complexity of function words in Chinese. Since function words in Chinese can be used flexibly and have varying functions, children may have difficulty considering them as dependable and consistent cues to learn verbs. One theoretical point even raises that Chinese may not have syntactic structures independent of semantics (e.g., Shen, 2018). If semantic and pragmatic cues are lacking in experimental tasks, relying solely on syntactic cues becomes more challenging.

However, such results did not necessarily mean that young Chinese learners acquired verbs later than English or French learners. Chinese‐speaking children have approximately the same number of nouns and verbs in their vocabulary, while English‐speaking children have a significantly higher proportion of nouns compared to verbs (Tardif, 2006). Furthermore, recent evidence revealed that very young Mandarin‐speaking toddlers are able to use syntactic cues in learning novel or familiar verbs. For example, Zhu et al. (2022) found that 17‐month‐old Mandarin‐speaking infants fixated more on a transitive scene than on a reflexive scene, when hearing NP‐V‐NP sentences with unfamiliar verbs but did not show the preference when hearing NP‐NP‐V sentences. Moreover, 19‐month‐old Mandarin‐speakers could also utilize word order (V‐*le* NP or NP V‐*le*) in differentiating familiar and even novel unaccusative and unergative verbs (Wang et al., in press). Considering that the unaccusative–unergative distinction is acquired very late by second‐language learners (Yuan, 1999), the fact that infants show an early ability to use the word order cues to differentiate the two subgroups of intransitive verbs suggests young Mandarin learners are not disadvantaged in verb learning. Young Mandarin learners may make use of many resources in verb acquisition other than function words.

Young Mandarin learners showed early utilizing of word order cues (e.g., Wang 2019; Zhu et al., 2022) and early understanding of function words in a familiar event (e.g., Chen et al., 2022) but exhibited comparatively later knowledge of the use of function words to learn novel verbs (e.g., Ma et al., 2019, and the present study). Taking into account the different experiment tasks and response requirement (looking preference versus explicit pointing) in these studies, the aforementioned discrepancy suggests that there could potentially be a significant development from using syntactic cues to comprehend familiar or even novel verbs to using syntactic cues to determine if a novel word functions as a verb. Therefore, the current study expands our understanding of syntactic bootstrapping in verb learning by demonstrating a comparatively later development (from 4 to 5 year‐olds) in the use of function words to learn novel verbs, despite the early understanding of function words among young Mandarin speakers.

### Can Mandarin‐speaking children generalize newly learned verbs to new morphosyntactic markers?

The present study first examined whether Mandarin‐speaking children could extend the newly learned verbs across different morphosyntactic markers. Although children can produce and understand certain verbs from the age of 10 months, such as “down” and “up” (Gentner, 1982), it does not guarantee full master of the meaning of these verbs. Young children tend to use certain words only in particular situations, but not in appropriate different situations (Bowerman, 1980). By examining children's ability of verbs extension, it is helpful for us to understand children's interpreting of new verbs. The results from Experiments 2 and 3 demonstrated that only 6‐year‐olds could recognize the newly learned verbs with a non‐verb‐biased/noun‐biased marker “*shì*.” However, 5‐year‐olds could recognize the newly learned verbs with a different marker “*le*” but not with a noun‐biased maker “*shì*.” The discrepancy for 5‐year‐olds in the two experiments might imply that 5‐year‐olds could not be very sure about the meaning of new words through learning from a sentence with the presence of verb marker “*zài*.” Therefore, their interpreting of new words might be context‐susceptible. To conclude, Mandarin‐speaking children could recognize the newly learned verbs with different morphosyntactic markers, but it depended on the bootstrapping of the cues in the new sentence.

### Multiple cues scaffold Mandarin‐speaking children's verb learning

Besides the linguistic cues that may scaffold toddlers' verb learning, there are many extralinguistic factors that affect the toddlers' verb learning, such as the objects used in the events (e.g., Haryu et al., 2011; Imai et al., 2005b) and the complexity of events (Childers et al., 2017; Scott & Fisher, 2012). We applied unfamiliar and dissimilar objects in this study to reduce the scaffolding of the familiarity and similarity of objects. Therefore, we could examine the role of function word more purely. However, we did not differentiate between whole‐body actions and complex actions involving hands. The actions we used might fall between the whole‐body actions and complex actions, mainly using hands and arms to initiate the motion of the objects. As verb learning might be better for whole‐body actions than complex actions (Scott & Fisher, 2012), the action type could be another important factor to be controlled or systematically manipulated in further studies.

Due to the complexity of verb learning, the Emergentist Coalition Model (ECM) proposed that children rely on multiple cues in early verb learning, such as perceptual, social, and linguistic cues (Hollich et al., 2000). Initially, children are more sensitive to perceptual cues in early verb learning and may attend to the appearance of objects during verb learning (Kersten & Smith, 2002). Then, children rely on social and linguistic cues more heavily (Brandone et al., 2007). Given the complexity involved in the process of verb learning, future studies could explore other effective cues for Mandarin learners to learn verbs as well as their integration.

In summary, the current study has revealed a new finding suggesting that the ability to use function words to learn new verbs can be developed between the ages of 4 and 5 years, thus providing a more complete understanding of the development. Additionally, the study presented initial evidence that 5‐year‐old Mandarin learners use syntactic cues, such as function words, to dynamically adjust their understanding of new words. Thus 5‐year‐olds who had encoded a new word as a verb during the learning phase might be at a loss to interpret it in a sentence with a non‐verb‐biased marker. Since function words in Chinese can be used flexibly and have varying functions, it is common for children to encounter a new verb across different function words. The findings reveal context dependence in children's representation of verbs even after they have learned to use function words to learn new verbs.

## CONCLUSION

The study showed the development of using function word “*zài*” to learn novel verbs among 3‐ to 6‐year‐old Mandarin‐speaking children. First, the ability of using the function word “*zài*” to determine a novel word as a verb but not a noun appears between the ages of 4 and 5 years. Second, 5‐year‐old Mandarin‐speaking children can use the function word “*zài*” to identify novel verbs and generalize the new verb with verb‐biased markers. Third, 6‐year‐old children have a relatively robust understanding of newly learned verbs because they can recognize the newly learned verbs even preceded by a non‐verb‐biased marker.

## CONFLICT OF INTEREST STATEMENT

The authors report there are no competing interests to declare.

## ETHICS STATEMENT

This study obtained ethics approval from the Research Ethics Committee of Zhejiang Normal University. The children were included after their parents or legal guardians gave oral consent to participate in the study.

## Data Availability

All data used for this study are stored on an Open Science Framework (OSF) page, see https://osf.io/uc89g/

## References

[pchj710-bib-0001] Bowerman, M. (1980). The structure and origin of semantic categories in the language learning child. In M. L. Foster & S. H. Brandes (Eds.), Symbol as a sense: New approaches to the analysis of meaning. Academic Press.

[pchj710-bib-0002] Brandone, A. C. , Pence, K. L. , Golinkoff, R. M. , & Hirsh‐Pasek, K. (2007). Action speaks louder than words: Young children differentially weight perceptual, social, and linguistic cues to learn verbs. Child Development, 78(4), 1322–1342. 10.1111/j.1467-8624.2007.01068.x 17650141

[pchj710-bib-0003] Chen, J. , & Shirai, Y. (2010). The development of aspectual marking in child mandarin Chinese. Applied PsychoLinguistics, 31(1), 1–28. 10.1017/S0142716409990257

[pchj710-bib-0004] Chen, L. , An, S. , Dai, H. , & He, X. (2022). Use of aspect markers by mandarin‐speaking children with high‐functioning autism plus language impairment and children with developmental language disorder. Journal of Communication Disorders, 99, 106245. 10.1016/j.jcomdis.2022.106245 35839538

[pchj710-bib-0005] Chen, L. , & Durrleman, S. (2022). Comprehension of mandarin aspect markers by preschool children with and without developmental language disorder. Frontiers in Psychology, 13, 839951. 10.3389/fpsyg.2022.839951 35572330 PMC9097452

[pchj710-bib-0006] Childers, J. B. , Paik, J. H. , Flores, M. , Lai, G. , & Dolan, M. (2017). Does variability across events affect verb learning in English, mandarin, and Korean? Cognitive Science, 41(suppl. 4), 808–830. 10.1111/cogs.12398 27457679 PMC5266742

[pchj710-bib-0007] de Carvalho, A. , Crimon, C. , Barrault, A. , Trueswell, J. , & Christophe, A. (2021). ‘Look! It is not a bamoule!’: 18‐ and 24‐month‐olds can use negative sentences to constrain their interpretation of novel word meanings. Developmental Science, 24(4), e13085. 10.1111/desc.13085 33484223 PMC8282655

[pchj710-bib-0008] de Carvalho, A. , He, A. X. , Lidz, J. , & Christophe, A. (2019). Prosody and function words cue the acquisition of word meanings in 18‐month‐old infants. Psychological Science, 30(3), 319–332. 10.1177/0956797618814131 30668928

[pchj710-bib-0009] Forbes, J. N. , & Poulin‐Dubois, D. (1997). Representational change in young children's understanding of familiar verb meaning. Journal of Child Language, 24(2), 389–406. 10.1017/S0305000997003127 9308424

[pchj710-bib-0010] Gao, S. (2015). Grammaticalization and acquisition order of "zai". In S. Gao (Ed.), A study on the acquisition sequence of Chinese concurrent function words based on grammaticalization theory (pp. 32–53). China Social Sciences Press (in Chinese).

[pchj710-bib-0011] Gentner, D. (1982). Why nouns are learned before verbs: Linguistic relativity versus natural partitioning. In S. Kuczaj (Ed.), Language development: Language, cognition, and culture (pp. 301–334). Erlbaum.

[pchj710-bib-0012] Gertner, Y. , Fisher, C. , & Eisengart, J. (2006). Learning words and rules: Abstract knowledge of word order in early sentence comprehension. Psychological Science, 17(8), 684–691. 10.1111/j.1467-9280.2006.01767.x 16913951

[pchj710-bib-0013] Gleitman, L. (1990). The structural sources of verb meanings. Language Acquisition, 1(1), 3–55. 10.1207/s15327817la0101_2

[pchj710-bib-0014] Haryu, E. , Imai, M. , & Okada, H. (2011). Object similarity bootstraps young children to action‐based verb extension. Child Development, 82(2), 674–686. 10.1111/j.1467-8624.2010.01567.x 21410924

[pchj710-bib-0015] He, A. X. , & Lidz, J. (2017). Verb learning in 14‐and 18‐month‐old English‐learning infants. Language Learning and Development, 13(3), 335–356. 10.1080/15475441.2017.1285238

[pchj710-bib-0016] Hollich, G. J. , Hirsh‐Pasek, K. , Golinkoff, R. M. , Brand, R. J. , Brown, E. , Chung, H. L. , Hennon, E. , & Rocroi, C. (2000). Breaking the language barrier: An emergentist coalition model for the origins of word learning. Monographs of the Society for Research in Child Development, 65(3), v–135. 10.1111/1540-5834.00090 12467096

[pchj710-bib-0017] Imai, E. H. , Haryu, E. , & Okada, H. (2005b). The role of object labels and familiarity in Japanese children's verb learning. In S. Watanabe (Ed.), CARLS series of advanced study of logic and sensibility (pp. 177–190). Keio University Press.

[pchj710-bib-0018] Imai, M. , Haryu, E. , & Okada, H. (2005a). Mapping novel nouns and verbs onto dynamic action events: Are verb meanings easier to learn than noun meanings for Japanese children? Child Development, 76(2), 340–355. 10.1111/j.1467-8624.2005.00849.x 15784086

[pchj710-bib-0019] Imai, M. , Li, L. , Haryu, E. , Okada, H. , Hirsh‐Pasek, K. , Golinkoff, R. M. , & Shigematsu, J. (2008). Novel noun and verb learning in Chinese‐, English‐, and Japanese‐speaking children. Child Development, 79(4), 979–1000. 10.1111/j.1467-8624.2008.01171.x 18717902

[pchj710-bib-0020] Kersten, A. W. , & Smith, L. B. (2002). Attention to novel objects during verb learning. Child Development, 73(1), 93–109. 10.1111/1467-8624.00394 14717246

[pchj710-bib-0021] Klein, W. , Li, P. , & Hendriks, H. (2000). Aspect and assertion in mandarin Chinese. Natural Language & Linguistic Theory, 18(4), 723–770. 10.1023/A:1006411825993

[pchj710-bib-0022] Kline, M. , & Demuth, K. (2014). Syntactic generalization with novel intransitive verbs. Journal of Child Language, 41(3), 543–574. 10.1017/S0305000913000068 23552211

[pchj710-bib-0023] Lee, J. N. , & Naigles, L. R. (2005). The input to verb learning in mandarin Chinese: A role for syntactic bootstrapping. Developmental Psychology, 41(3), 529–540. 10.1037/0012-1649.41.3.529 15910160

[pchj710-bib-0024] Li, P. , & Bowerman, M. (1998). The acquisition of lexical and grammatical aspect in Chinese. First Language, 18(54), 311–350. 10.1177/014272379801805404

[pchj710-bib-0025] Ma, W. , Zhou, P. , & Golinkoff, R. M. (2020). Young mandarin learners use function words to distinguish between nouns and verbs. Developmental Science, 23(5), e12927. 10.1111/desc.12927 31793739

[pchj710-bib-0026] Ma, W. , Zhou, P. , Golinkoff, R. M. , Lee, J. , & Hirsh‐Pasek, K. (2019). Syntactic cues to the noun and verb distinction in mandarin child‐directed speech. First Language, 39(4), 433–461. 10.1177/0142723719845175

[pchj710-bib-0027] Maguire, M. J. , Hennon, E. a. , Hirsh‐Pasek, K. , Golinkoff, R. M. , Slutzky, C. B. , & Sootsman, J. (2002). Mapping words to actions and events: How do 18‐month‐olds learn a verb? In B. Skarabela , S. Fish , & A. Do (Eds.), Proceedings of the 27th annual Boston University conference on language (pp. 371–382). Cascadilla Press.

[pchj710-bib-0028] Maguire, M. J. , Hirsh‐Pasek, K. , Golinkoff, R. M. , & Brandone, A. C. (2008). Focusing on the relation: Fewer exemplars facilitate children's initial verb learning and extension. Developmental Science, 11(4), 628–634. 10.1111/j.1467-7687.2008.00707.x 18576970

[pchj710-bib-0029] Naigles, L. (1990). Children use syntax to learn verb meanings. Journal of Child Language, 17(2), 357–374. 10.1017/S0305000900013817 2380274

[pchj710-bib-0030] Naigles, L. R. , Bavin, E. L. , & Smith, M. A. (2005). Toddlers recognize verbs in novel situations and sentences. Developmental Science, 8(5), 424–431. 10.1111/j.1467-7687.2005.00431.x 16048515

[pchj710-bib-0031] Scott, R. M. , & Fisher, C. (2012). 2.5‐year‐olds use cross‐situational consistency to learn verbs under referential uncertainty. Cognition, 122(2), 163–180. 10.1016/j.cognition.2011.10.010 22104489 PMC3246112

[pchj710-bib-0032] Shen, J. X. (2018). Problems caused by modelling Chinese grammar on subject and predicate. Journal of Foreign Languages, 41(6), 2–15. (in Chinese).

[pchj710-bib-0033] Su, Y. , & Naigles, L. R. (2021). Comprehension of grammatical aspect markers *le* and zai in a diverse sample of mandarin‐exposed preschool children with autism spectrum disorder. Reading and Writing, 1–24, 1369–1392. 10.1007/s11145-021-10214-w

[pchj710-bib-0034] Tardif, T. (1996). Nouns are not always learned before verbs: Evidence from mandarin speakers' early vocabularies. Developmental Psychology, 32(3), 492–504. 10.1037/0012-1649.32.3.492

[pchj710-bib-0035] Tardif, T. (2006). But are they really verbs? Chinese words for action. In K. Hirsh‐Pasek & R. M. Golinkoff (Eds.), Action meets world: How children learn verbs (Vol. 1, pp. 477–498). Oxford University Press.

[pchj710-bib-0036] Tatsumi, T. , Ambridge, B. , & Pine, J. (2018). Disentangling effects of input frequency and morphophonological complexity on Children's Acquisition of Verb Inflection: An elicited production study of Japanese. Cognitive Science, 42(S2), 555–577. 10.1111/cogs.12554 29023860 PMC6001619

[pchj710-bib-0037] Theakston, A. , Lieven, E. , Pine, J. , & Rowland, C. (2002). Going, going, gone: The acquisition of the verb ‘go’. Journal of Child Language, 29(4), 783–811. 10.1017/s030500090200538x 12471973

[pchj710-bib-0038] Wakefield, E. M. , Hall, C. , James, K. H. , & Goldin‐Meadow, S. (2018). Gesture for generalization: Gesture facilitates flexible learning of words for actions on objects. Developmental Science, 21(5), e12656. 10.1111/desc.12656 29542238

[pchj710-bib-0039] Wang, H. L. (2019). A study on the acquisition order of the “Shi” sentences of Chinese learners in Malawi. Master's thesis. Zhejiang Normal University, Jinhua (in Chinese).

[pchj710-bib-0040] Wang, Z. Q. , Yang, X. L. , & Shi, R. S. (in press). Mandarin‐learning 19‐month‐old toddlers' sensitivity to word order cues that differentiate unaccusative and unergative verbs. Journal of Child Language, (published online), 1–22. 10.1017/S0305000922000629 36458337

[pchj710-bib-0041] Yang, X. , Shi, R. , & Xu, K. (2018). Grammatical aspect in early child mandarin: Evidence from a preferential looking experiment. Journal of Psycholinguistic Research, 47(6), 1301–1320. 10.1007/s10936-018-9590-7 29961248

[pchj710-bib-0042] Yuan, B. (1999). Acquiring the unaccusative/unergative distinction in a second language: Evidence from English‐speaking learners of L2 Chinese. Linguistics, 37(2), 275–296. 10.1515/ling.37.2.275

[pchj710-bib-0043] Yuan, S. , & Fisher, C. (2006). ‘Really? He blicked the cat?’: Two‐year‐olds learn distributional facts about verbs in the absence of a referential context. In Proceedings of the 30th annual Boston University conference on language development (pp. 689–700). Cascadilla Press.

[pchj710-bib-0044] Yuan, S. , & Fisher, C. (2009). ‘Really? She blicked the baby?’ Two‐year‐olds learn combinatorial facts about verbs by listening. Psychological Science, 20(5), 619–626. 10.1111/j.1467-9280.2009.02341.x 19476591 PMC3989287

[pchj710-bib-0045] Yuan, S. , Fisher, C. , & Snedeker, J. (2012). Counting the nouns: Simple structural cues to verb meaning. Child Development, 83(4), 1382–1399. 10.1111/j.1467-8624.2012.01783.x 22616898

[pchj710-bib-0046] Zhou, P. , Crain, S. , & Zhan, L. (2014). Grammatical aspect and event recognition in children's online sentence comprehension. Cognition, 133(1), 262–276. 10.1016/j.cognition.2014.06.018 25061759

[pchj710-bib-0047] Zhou, P. , & Ma, W. (2018). Children's use of morphological cues in real‐time event representation. Journal of Psycholinguistic Research, 47(1), 241–260. 10.1007/s10936-017-9530-y 29105015

[pchj710-bib-0048] Zhu, J. , Franck, J. , Rizzi, L. , & Gavarro, A. (2022). Do infants have abstract grammatical knowledge of word order at 17 months? Evidence from mandarin Chinese. Journal of Child Language, 49(1), 60–79. 10.1017/S0305000920000756 33550998

